# Comprehensive characterization of gastrointestinal microbiota dysbiosis in patients with refractory *Helicobacter pylori* infection

**DOI:** 10.1128/msystems.01090-25

**Published:** 2025-09-30

**Authors:** Yu Li, Xinbo Xu, Chao Peng, Yichen Liu, Nonghua Lu, Yin Zhu, Cong He

**Affiliations:** 1 Department of Gastroenterology, The First Affiliated Hospital, Jiangxi Medical College, Nanchang University 47861, Nanchang, Jiangxi, China; 2 Jiangxi Provincial Key Laboratory of Digestive Diseases, The First Affiliated Hospital, Jiangxi Medical College, Nanchang University 47861, Nanchang, Jiangxi, China; 3 HuanKui Academy, Nanchang University 47861, Nanchang, Jiangxi, China; Institut de Recherche pour le Developpement Delegation Regionale Occitanie, Montpellier, France

**Keywords:** *Helicobacter pylori*, eradication failure, gastrointestinal microbiota, 16S rRNA sequencing

## Abstract

While repeated *Helicobacter pylori* eradication attempts are increasingly common due to antimicrobial resistance, the compounding effects of multiple antibiotic courses on gastrointestinal microbiota remain unquantified. We enrolled 32 treatment-naïve patients and 84 patients with refractory *H. pylori* infections, defined as failure of at least two prior eradication attempts. Antibiotic susceptibility testing was conducted for refractory *H. pylori* isolates to assess resistance patterns. Gastric mucosal biopsies and fecal samples were obtained for 16S rRNA gene sequencing. Antimicrobial susceptibility testing revealed resistance rates of 96.55% to metronidazole, 77.59% to clarithromycin, 56.90% to levofloxacin, 5.17% to rifampicin, 1.72% to tetracycline, and 0% to amoxicillin and furazolidone. The rates of dual drug and multidrug resistance were 27.59% and 55.17%, respectively. Refractory patients exhibited significant gastric microbiota restructuring, marked by reduced alpha diversity. The refractory group had higher concentrations of pathogens like *Pseudomonas* and *Burkholderia* and lower relative abundances of beneficial bacteria, including *Bifidobacterium*, *Blautia*, and *Roseburia*. Levofloxacin-resistant individuals exhibited an amplified abundance of pathogenic *Veillonella*. The gastric microbial diversity was higher in clarithromycin-resistant patients, with the enrichment of *Micromonospora*. Compared with the single-resistant group, pathogens, such as *Veillonella* and *Peptostreptococcus*, were increased in the multidrug-resistant group. We also observed perturbations of gut microbiota in refractory *H. pylori*-infected patients, with the predominance of *Streptococcus* and depletion of *Bacteroides*. Our findings establish that refractory *H. pylori* infection induces a disruption of gastrointestinal microbiota, highlighting the importance of optimizing first-line regimens to prevent retreatment cycles and the potential benefit of microbiota-modulating strategies in refractory *H. pylori* management.

**IMPORTANCE:**

Previous research has demonstrated that *H. pylori* eradication therapies can transiently alter gut microbiota. However, the long-term consequences of repeated antibiotic treatments in refractory infections remain unexplored. In this study, we link failed eradication attempts to persistent gastrointestinal dysbiosis, characterized by increased *Pseudomonas* and antibiotic-resistant *Veillonella*, alongside depletion of beneficial bacteria. In addition, we also demonstrate distinct gastric microbiota structure in patients with different antibiotic resistance patterns. Our findings showed distinct microbial dysbiosis after repeated eradication attempts, highlighting the need to explore microbiota-modulating approaches in future clinical trials.

## INTRODUCTION

*Helicobacter pylori* infects nearly half of the global population and is a key etiological factor in multiple gastric pathologies, such as chronic gastritis, peptic ulcers, and gastric cancer. Consequently, *H. pylori* eradication has been widely advocated to reduce the incidence of these conditions (1, 2). Standard treatment regimens rely on combination therapies (dual, triple, or quadruple), typically comprising a proton-pump inhibitor (PPI) alongside antibiotics like clarithromycin, amoxicillin, or metronidazole (3). However, the rising prevalence of antibiotic resistance has significantly compromised treatment efficacy, leading to an increasing number of patients with poor clinical outcomes (4, 5). For instance, clarithromycin-based triple therapy, once a highly effective first-line approach, now exhibits an eradication rate of less than 80% (2, 6). Although levofloxacin-based triple therapy and bismuth-containing quadruple therapy serve as common second-line options, treatment failure still occurs in 10%–20% of cases (7, 8). Patients who do not respond to at least two eradication attempts are defined as having refractory *H. pylori* infection (9).

Recent advancements in sequencing technologies and analytical methodologies have elucidated the profound effects of *H. pylori* eradication on gastrointestinal microbiota (10, 11). Contemporary studies reveal both enhanced microbial diversity and fundamental restructuring of gastric microbial communities following eradication therapy (12, 13). With respect to gut microbiota, mounting studies have documented transient perturbations in fecal microbiota diversity accompanied by an elevation in antibiotic resistance genes during the initial post-treatment phase (14, 15). However, longitudinal investigations have revealed that the gastrointestinal microbiota homeostasis can be effectively restored over an extended period after successful eradication therapy (15, 16). To date, the effects of multiple eradication therapies on gastrointestinal microbiota are still unclear. Giving the rapid rise in antimicrobial resistance, it is crucial to understand the gastrointestinal microbiota profile in refractory *H. pylori* infections and identify the major bacteria associated with drug resistance and eradication failure.

In this study, we employed 16S rRNA sequencing to characterize the gastrointestinal microbiota composition and identify dysbiotic patterns in patients with refractory *H. pylori* infection. Following multiple *H. pylori* eradication courses, a notable reduction in both gastric and gut bacterial diversity was observed, accompanied by significant alterations in microbial community structure. Furthermore, we conducted comprehensive comparative analyses to evaluate gastric microbiota composition across distinct antibiotic resistance profiles, including levofloxacin-sensitive versus levofloxacin-resistant group, clarithromycin-sensitive versus clarithromycin-resistant group, as well as single-drug resistant versus multidrug-resistant populations. Our findings delineate the persistent microbial ecosystem dysbiosis following repeated eradication treatments and provide novel insights into developing microbiota-based therapeutic strategies for managing refractory *H. pylori* infection.

## MATERIALS AND METHODS

### Patients and sample collection

A total of 116 patients were enrolled from The First Affiliated Hospital of Nanchang University and divided into two groups: (i) Group S: treatment-naïve patients with *H. pylori* infection who achieved successful eradication after the initial standard therapy in a previous randomized double-blind placebo-controlled trial (17), as confirmed by a negative result on ^13^C urea breath test; (ii) Group F: patients who have failed to eradicate *H. pylori* for two or more times and had discontinued all eradication medications for at least 6 months. The inclusion criteria were (i) age 18–70 years; (ii) no antibiotic, probiotic, proton-pump inhibitor (PPI), synbiotic, bismuth, hormonal, or immunosuppressant use within the preceding 6 months. The gastric mucosa biopsies and fecal samples were collected from Group S (baseline samples obtained prior to any therapeutic intervention) and Group F (samples collected prior to salvage therapy, with at least a 6-month washout period since their last eradication treatment), immediately frozen at −80°C, and stored until DNA extraction. Prior to enrollment, written informed consent was secured from all patients. The study protocol received approval from the Ethics Committee of The First Affiliated Hospital of Nanchang University (2019055) and was registered with the Chinese Clinical Trial Registry (ChiCTR1900023008).

### Relevant definition

Refractory *H. pylori* infection is defined as a positive non-serological test (e.g., urea breath test, stool antigen test, or gastroscopy-based detection) persisting ≥4 weeks after completion of one or more courses of guideline-recommended first-line eradication therapy, with no recent use of medications that could compromise test sensitivity (9).

### *H. pylori* culture and antibiotic susceptibility test

Gastric mucosal biopsy specimens obtained from Group F were preserved in brain heart infusion broth (Oxoid, Basingstoke, UK) containing 20% glycerol and stored at −80°C until processing. Following homogenization, the specimens were cultured on Campylobacter-selective agar plates (Oxoid, Basingstoke, UK) enriched with 5% defibrinated sheep blood (Bio-Kont, Zhejiang, China) and antimicrobial supplements, including 2.5 mg/L vancomycin, 3 mg/L trimethoprim, 2 mg/L polymyxin B, and 2 mg/L amphotericin B (Duly Biotech, Nanjing, China). All cultures were maintained at 37°C under microaerobic conditions (10% CO_2_, 5% O_2_, and 85% N_2_) for a maximum duration of 5 days.

Antibiotic susceptibility testing was conducted using E-test strips to determine the minimum inhibitory concentrations (MICs) for metronidazole, clarithromycin, levofloxacin, rifampicin, tetracycline, and amoxicillin. Furazolidone susceptibility was assessed via the Kirby-Bauer disk diffusion method. Resistance thresholds were defined as follows: MIC > 8 mg/L for metronidazole, ≥1 mg/L for clarithromycin, >2 mg/L for levofloxacin, >1 mg/L for rifampicin, ≥2 mg/L for tetracycline, >0.125 mg/L for amoxicillin, and an inhibition zone diameter ≤7 mm for furazolidone. *H. pylori* culture and antibiotic susceptibility testing were performed at the Institute of Gastroenterology and Hepatology, the First Affiliated Hospital of Nanchang University (18).

### DNA extraction and 16s rRNA gene amplification

Genomic DNA was extracted from all samples using the OMEGA Soil DNA Kit (M5635-02; Omega Bio-Tek, Norcross, GA, USA) following the manufacturer’s protocol, then stored at −20°C for subsequent analysis. DNA concentration and purity were determined using a NanoDrop NC2000 spectrophotometer (Thermo Fisher Scientific, Waltham, MA, USA), with integrity confirmed by 1% agarose gel electrophoresis. For microbial community analysis, the V3–V4 hypervariable regions of the 16S rRNA gene were PCR-amplified using the universal bacterial primers 338F (5′-ACTCCTACGGGAGGCAGCA-3′) and 806R (5′-GGACTACHVGGGTWTCTAAT-3′).

### Library construction and sequencing

PCR amplicons were purified using Vazyme VAHTSTM DNA Clean Beads (Vazyme Biotech, Nanjing, China) and quantified with the Quant-iT PicoGreen dsDNA Assay Kit (Thermo Fisher Scientific, Carlsbad, CA, USA). Following individual quantification, equimolar amounts of amplicons were pooled for paired-end sequencing (2 × 250 bp) on an Illumina NovaSeq platform (NovaSeq 6000 SP Reagent Kit, 500 cycles; Shanghai Personal Biotechnology Co., Ltd, Shanghai, China).

### Bioinformatic analysis

Microbiome bioinformatics analysis was conducted in QIIME2 (version 2019.4), following established pipelines with minor modifications (19). Raw sequencing reads were demultiplexed using the demux plugin, followed by primer removal with cutadapt (20). Quality filtering, denoising, read merging, and chimera removal were performed using DADA2 (21). High-quality sequences were clustered into operational taxonomic units (OTUs) at 97% similarity threshold via the UPARSE pipeline (22). Taxonomic assignment of representative OTUs was carried out using the RDP classifier (version 2.2) against the SILVA database (version 138.1), implementing a naive Bayesian classification approach.

Microbial alpha diversity was assessed using Chao1 (richness), Shannon (diversity), and observed species (Sobs) indices. Beta diversity was evaluated through weighted UniFrac and Bray-Curtis distance metrics, with principal coordinates analysis (PCoA) visualization implemented via the Vegan package in R. For differential abundance analysis, we employed MaAsLin2 (Multivariate Association with Linear Models) to account for confounding variables in groups with unmatched baseline characteristics, while Wilcoxon rank-sum tests were applied to matched groups (23). Functional profiling of the gastric microbiota was performed using PICRUSt2 to predict metagenomic content, with subsequent identification of differentially abundant KEGG pathways determined by Wilcoxon rank-sum tests.

### Statistical analysis

Demographic characteristics were analyzed using Student’s *t*-tests in SPSS version 20.0 (IBM), with continuous variables expressed as mean ± standard deviation. Microbial alpha diversity comparisons employed Kruskal-Wallis tests, while beta diversity differences were assessed using PERMANOVA (adonis). Statistical significance was defined as *P* < 0.05 for all analyses.

## RESULTS

### Patient characteristics

Demographic characteristics of the study cohort were summarized in Table S1. A total of 116 patients were enrolled, comprising 32 treatment-naïve *H. pylori*-infected patients and 84 patients with refractory *H. pylori* infection. The age with significant difference was adjusted in the following analysis. After administering bismuth quadruple therapy, Group S achieved successful eradication. The antimicrobial susceptibility testing was conducted for Group F. Among the samples, metronidazole resistance was predominant (96.55%), followed by clarithromycin (77.59%) and levofloxacin (56.90%). The prevalence of dual and multidrug resistance among refractory cases was 27.59% and 55.17%, respectively. Among refractory *H. pylori-*infected patients, the most prevalent resistance pattern was triple resistance to levofloxacin, clarithromycin, and metronidazole (*n* = 28, 48.28%), followed by dual clarithromycin-metronidazole resistance (*n* = 14, 24.14%). Less frequent patterns included levofloxacin-metronidazole dual resistance (*n* = 2, 3.45%) and clarithromycin-rifampicin-metronidazole triple resistance (*n* = 2, 3.45%). Furthermore, no significant differences in age or gender distribution were observed between patients resistant and susceptible to levofloxacin and clarithromycin (Tables S2 to S4).

### The gastric microbial features in patients with refractory H*. pylori* infection compared to treatment-naïve individuals

**Fig 1:**
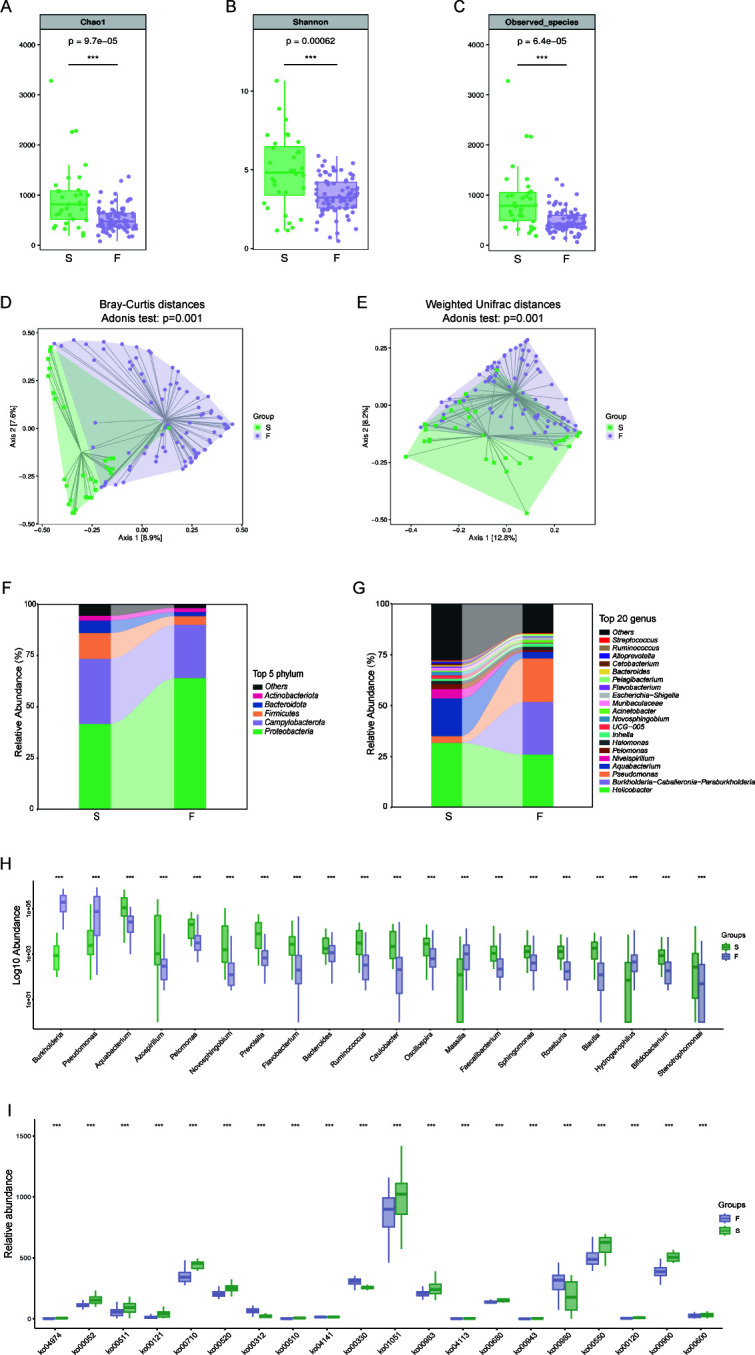
Comparative analysis revealed distinct gastric microbiota profiles between treatment-naïve and refractory *H. pylori* infections. Refractory cases exhibited significantly lower alpha diversity, as illustrated by Chao1 (**A**), Shannon (**B**), and sobs index (**C**). PCoA based on Bray-Curtis distances (**D**) and Weighted UniFrac distances (**E**) demonstrated pronounced structural differences. Relative proportions of the top 5 most abundant bacteria at the phylum level (**F**) and top 20 most abundant bacteria at the genus level (**G**). MaAsLin2 analysis identified differentially abundant taxa (**H**), while functional profiling revealed distinct KEGG pathways at level 3 (**I**) between groups. S, treatment-naïve patients with *H. pylori* infection; F, patients with refractory *H. pylori* infection; Sobs, number of observed operational taxonomic units; PCoA, principal coordinate analysis; MaAsLin2, multivariate association with linear models; KEGG, Kyoto Encyclopedia of Genes and Genomes. ***P < 0.001.

Comparative analysis revealed distinct differences in microbial diversity between groups. Alpha diversity metrics (Chao1, Shannon, and Sobs indices) demonstrated markedly reduced richness and diversity in Group F versus Group S mucosa (Fig. 1A through C, *P* = 0.000097 for Chao1 index, *P* = 0.00062 for Shannon index, *P* = 0.000064 for Sobs index). Beta diversity analysis revealed distinct clustering patterns, with principal coordinates analysis showing clear separation between groups (Fig. 1D and E, PERMANOVA, *P* = 0.001 for both Weighted UniFrac and Bray-Curtis distances), indicating substantial differences in microbiota composition between Group F and Group S.

To characterize specific microbial alterations, we analyzed bacterial colonization patterns and community composition. Proteobacteria, Campylobacterota, and Firmicutes emerged as the three most abundant phyla, displaying distinct distribution patterns between Group S and Group F (Fig. 1F). In refractory *H. pylori* infections, Proteobacteria predominated, comprising 63.94% of the microbiota. Although Firmicutes and Bacteroidetes exhibited declining trends, the Firmicutes/Bacteroidetes ratio was notably higher in Group F. At the genus level, *Helicobacter* and *Aquabacterium* were predominant in Group S, whereas *Helicobacter*, *Burkholderia–Caballeronia–Paraburkholderia*, and *Pseudomonas* collectively represented 70% of the bacterial composition in Group F. The relative abundances of *Burkholderia–Caballeronia–Paraburkholderia* and *Pseudomonas* was markedly elevated in Group F (25.87% and 21.39%, respectively) compared with Group S (0.16% and 3.20%, respectively) (Fig. 1G and H). Conversely, beneficial bacteria, including *Prevotella*, *Ruminococcus,* and *Bifidobacterium* were significantly depleted in Group F.

**Fig 2:**
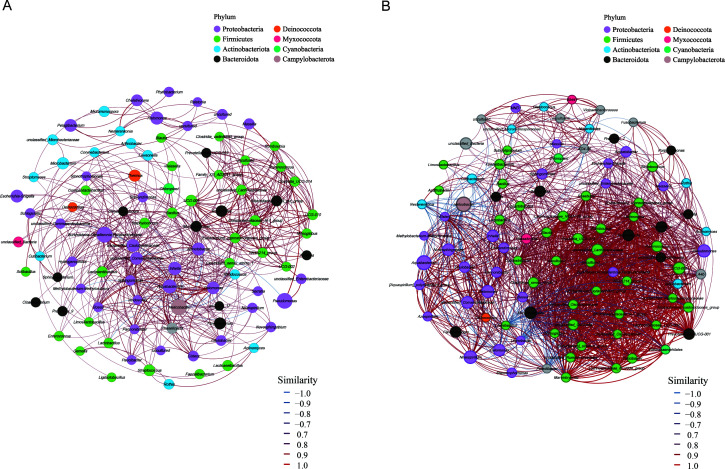
Co-occurrence network analysis demonstrates a fundamental restructuring of gastric microbial ecology in refractory *H. pylori* infections (**A**) compared with treatment-naïve cases (**B**). Nodes represent bacterial genera, with size scaled to relative abundance and color-coded by network module. Edge properties indicate ecological interactions: red for positive correlations, blue for negative correlations, and thickness proportional to correlation strength.

Functional metagenomic profiling was conducted through PICRUSt analysis of 16S rRNA gene sequencing data (Fig. 1I; Table S5). Patients with refractory *H. pylori* infection exhibited significant enrichment of metabolic pathways associated with beta-lactam resistance (ko00312). The gastric microbiome of patients in Group S was characterized by over-representation of drug metabolism—other enzymes (ko00983), secondary bile acid biosynthesis (ko00121), and amino sugar and nucleotide sugar metabolism (ko00520). These findings suggest the development of a microbial community with enhanced drug resistance potential following refractory *H. pylori* infection. To assess how refractory *H. pylori* infection alters microbial ecological relationships, we conducted co-occurrence network analysis of the gut microbiota. The gastric microbiota in Group F (Fig. 2A) demonstrated significantly fewer co-occurrence interactions compared with Group S (Fig. 2B). We observed that in Group F, *Helicobacter* demonstrated predominantly positive correlations with other gastric microbiota members, including *Streptococcus*, suggesting co-occurring interactions. Conversely, in Group S, *Helicobacter* exhibited significant negative correlations with co-existing bacterial species, such as *Lactobacillus*, *Faecalibacterium*, and *Sphingomonas*, indicating co-excluding interactions. Notably, *Burkholderia-Caballeronia-Paraburkholderia*, which was enriched in refractory *H. pylori* infection patients, exhibited co-occurrence relationships with *Sphingomonas*, *Aquabacterium*, and *Staphylococcus*. Microbial co-occurrence analysis revealed a significant positive correlation between *Blautia* and *Bacteroides* in Group S. On the contrary, pathogenic genera *Enterococcus* and *Escherichia-Shigella* demonstrated strong positive interrelationships, while exhibiting significant negative correlations with *Bacteroides* abundance. These findings suggest substantial alterations in microbial network topology associated with refractory *H. pylori* infection.

### Alterations of gastric microbiota in levofloxacin-resistant versus susceptible patients

**Fig 3:**
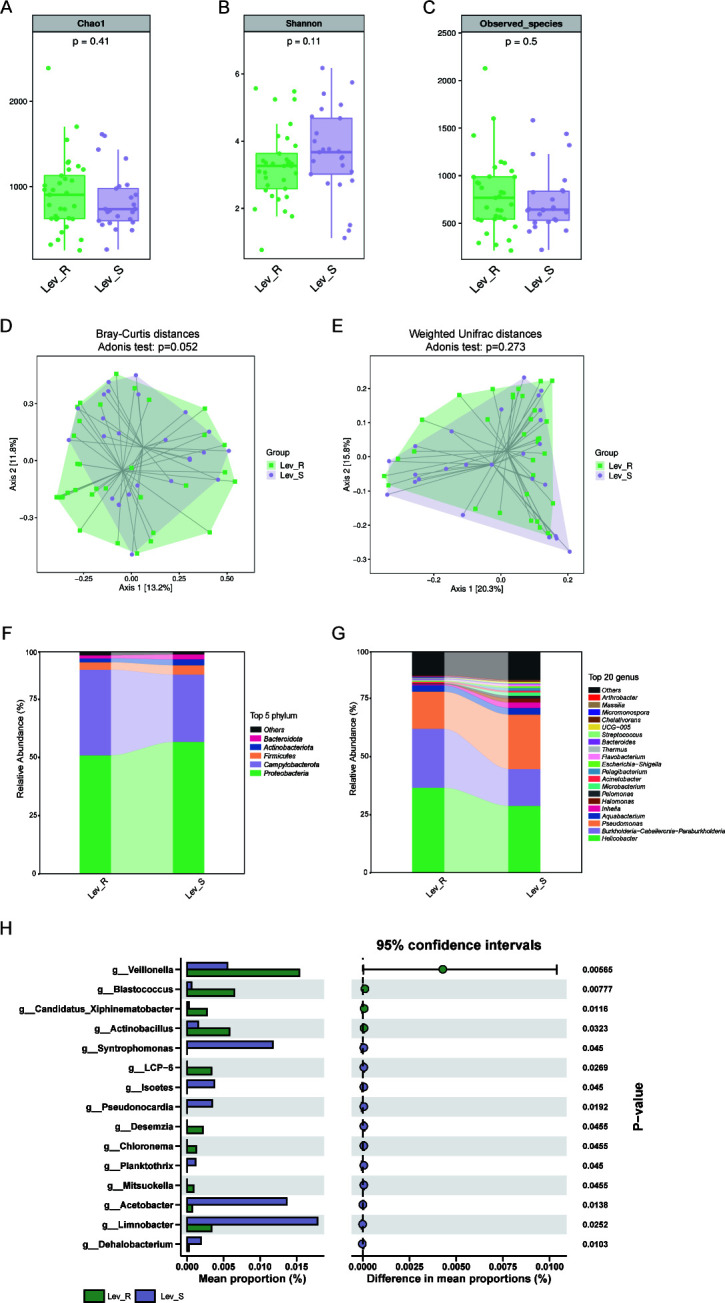
Changes in gastric microbiota in levofloxacin-resistant versus levofloxacin-susceptible patients. There was no significant difference in bacterial alpha diversity measured by Chao1 (**A**), Shannon (**B**), and Sobs index (**C**) between Lev_R and Lev_S. PCoA based on Bray-Curtis distances (**D**) and Weighted UniFrac distances (**E**) revealed no substantial divergence in community structure. Taxonomic composition, represented by the top 5 phyla (**F**) and top 20 genera (**G**), showed comparable distributions between groups. Quantitative comparison of relative abundances (**H**) further confirmed minimal intergroup variation, with mean proportions (%) and 95% confidence intervals indicating negligible differences. Lev_R, levofloxacin-resistant patients; Lev_S, levofloxacin-susceptible patients; Sobs, number of observed operational taxonomic units; PCoA, principal coordinate analysis.

Alpha diversity analysis revealed distinct microbial patterns between levofloxacin-resistant (Lev_R) and susceptible (Lev_S) patients, with Lev_R demonstrating higher richness (Chao1 and Sobs index) but lower diversity (Shannon index) (Fig. 3A through C). Beta diversity analysis, assessed through Bray-Curtis and Weighted UniFrac metrics and visualized via PCoA, revealed distinct clustering patterns. While Lev_R and Lev_S groups demonstrated measurable compositional differences, these were less pronounced than the robust separation observed between Group F and Group S (Fig. 3D and E). Taxonomic profiling demonstrated similar phylum-level composition between Lev_R and Lev_S, while genus-level analysis identified a significant increase in *Veillonella* and *Actinobacillus* abundance in Lev_R compared with Lev_S (Fig. 3F through H). These findings indicate specific microbial alterations associated with levofloxacin resistance.

### Alterations of gastric microbiota in clarithromycin-resistant versus clarithromycin-susceptible patients

**Fig 4:**
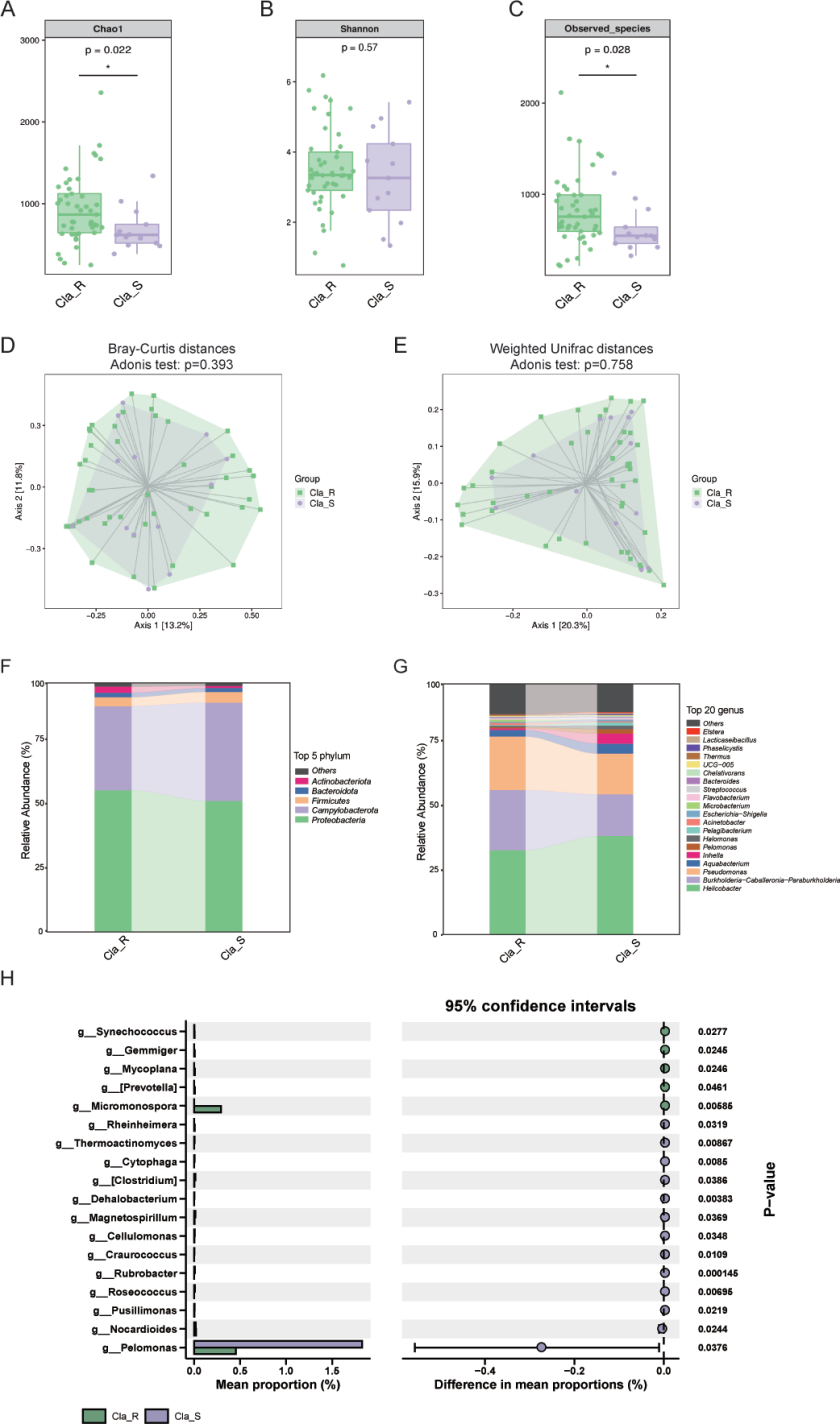
Variation of gastric microbiota between clarithromycin-resistant and clarithromycin-susceptible patients. Alpha diversity analysis demonstrated that Chao1 (**A**) and Sobs (**C**) were lower in Cla_R than Cla_S, while no significant difference was observed in Shannon (**B**). Beta diversity analysis indicated by PCoA plots of Bray-Curtis distances (**D**) and Weighted UniFrac distances (**E**) depicted not distinct clustering between Cla_R and Cla_S. The distinct distribution of the top 5 most abundant taxa in different groups at phylum (**F**) level and the top 20 most abundant taxa at genus (**G**) level. The Wilcoxon rank-sum test was performed to identify the significantly changed genera (**H**) between these two groups. Cla_R, clarithromycin-resistant patients; Cla_S, clarithromycin-susceptible patients; Sobs, number of observed operational taxonomic units; PCoA, principal coordinate analysis. *P < 0.05.

Microbial alpha diversity analysis denoted that richness calculated by Chao1 and Sobs index was prominently higher in patients resistant to clarithromycin (Cla_R) compared with susceptible (Cla_S) individuals, whereas Shannon index showed no significant intergroup differences (Fig. 4A through C). The PCoA demonstrated no distinct clustering between Cla_R and Cla_S, supported by non-significant PERMANOVA *P*-values for both distance metrics (Fig. 4D and E). Taxonomic analysis revealed comparable phylum-level composition between the groups (Fig. 4F). However, Cla_R patients exhibited increased relative abundance of *Micromonospora*, suggesting potential microbial markers associated with clarithromycin resistance (Fig. 4G and H).

### Alterations of gastric microbiota in single-drug versus multidrug-resistant patients

**Fig 5:**
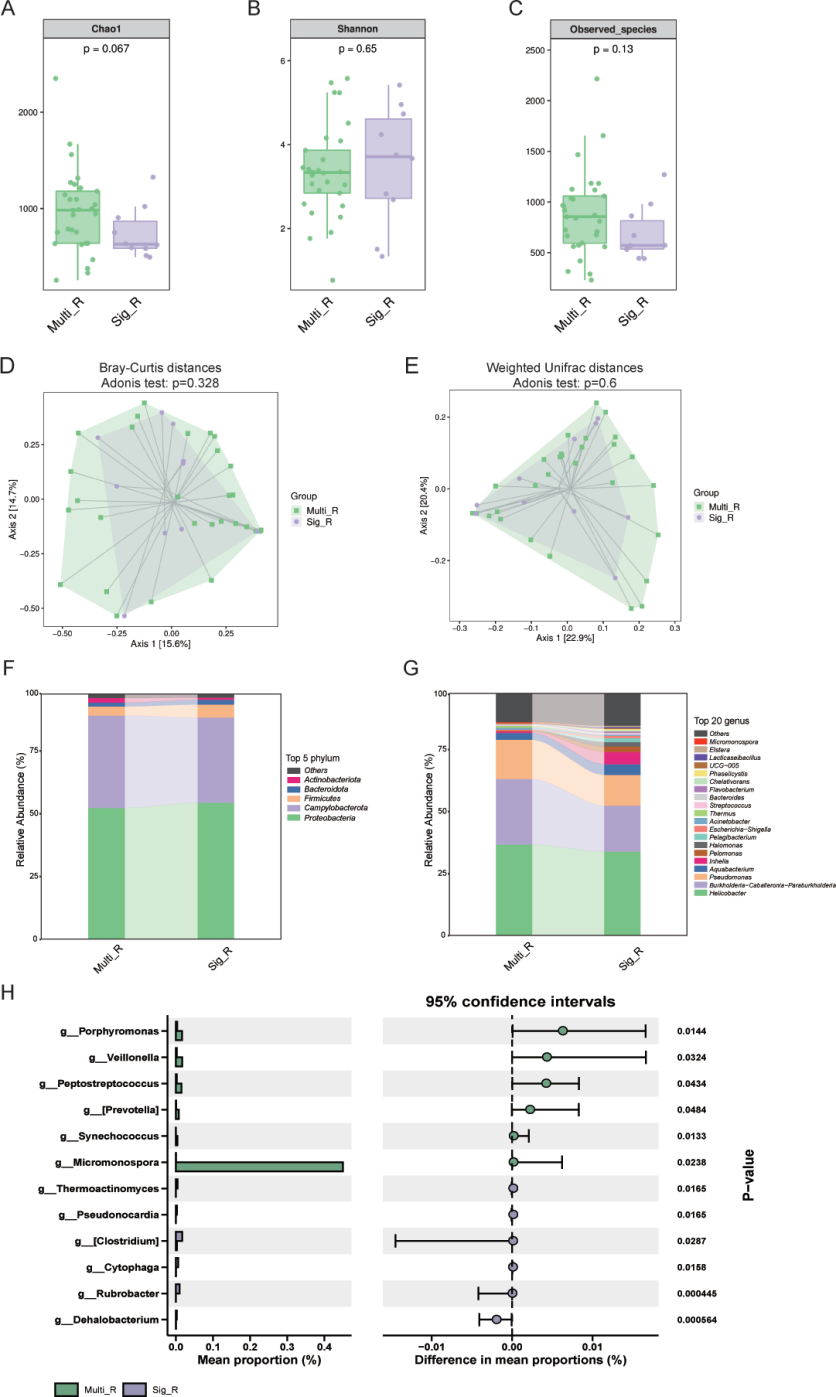
Changes in gastric microbiota in single-drug versus multidrug-resistant patients. There was no significant difference in microbial alpha diversity, as measured by Chao1 (**A**), Shannon (**B**), and Sobs index (**C**), between Multi_R and Sig_R. PCoA based on Bray-Curtis distances (**D**) and Weighted UniFrac distances (**E**) showed that the difference in microbiota structure between Multi_R and Sig_R was not significant. Relative abundance of the top 5 most abundant bacteria at the phylum level (**F**) and top 20 most abundant bacteria at the genus level (**G**). Comparison of the relative abundance of gastric microbiota between Multi_R and Sig_R (**H**). Multi_R, multidrug-resistant patients; Sig_R, single-drug-resistant patients; Sobs, number of observed operational taxonomic units; PCoA, principal coordinate analysis.

Alpha diversity analysis demonstrated no significant differences in microbial richness (Chao1, Sobs index) or diversity (Shannon index) between single-drug resistant (Sig_R) and multidrug-resistant (Multi_R, defined as resistant to three or more commonly used antibiotics) patients (Fig. 5A through C). Beta diversity assessment, visualized in PCoA, revealed no significant compositional differences between Sig_R and Multi_R (Fig. 5D and E). Proteobacteria was the most abundant phylum in both groups (Fig. 5F). At the genus level, multidrug-resistant patients exhibited increased abundance of *Micromonospora*, *Veillonella*, and *Peptostreptococcus*, coupled with reduced levels of *Dehalobacterium*, suggesting distinct microbial signatures associated with multidrug resistance profiles (Fig. 5G and H).

### Disturbance of gut microbiota afte**r** refractory *H. pylori* infection

**Fig 6:**
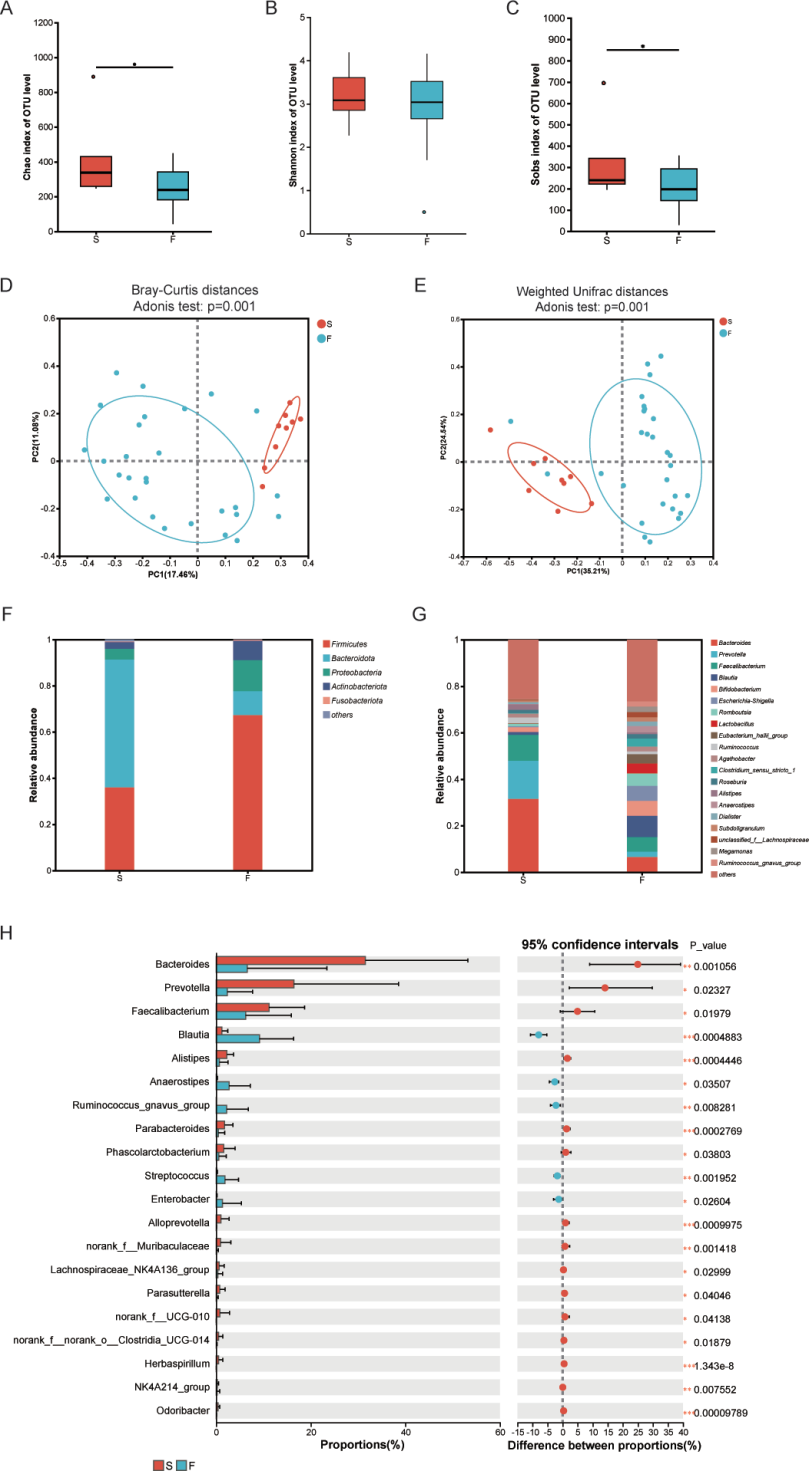
Variation of gut microbiota between treatment-naïve and refractory *H. pylori*-infected individuals. Alpha diversity metrics demonstrated that Chao1 (**A**) and Sobs (**C**) were lower in group F than group S, while no significant difference was observed in Shannon (**B**). Beta diversity analysis indicated by PCoA of Bray-Curtis distances (**D**) and Weighted UniFrac distances (**E**) demonstrated overlapping clustering patterns without clear separation between these two groups. The differential distribution of the top 5 most abundant taxa across different groups at phylum (**F**) level and the top 20 most abundant taxa at genus (**G**) level. Significant intergroup differences in genus-level abundance were determined using the Wilcoxon rank-sum test (**H**). S, treatment-naïve patients with *H. pylori* infection; F, patients with refractory *H. pylori* infection; Sobs, number of observed operational taxonomic units; PCoA, principal coordinate analysis. *P < 0.05.

While transient gut microbiota dysbiosis following *H. pylori* eradication has been documented, the cumulative effects of repeated antibiotic therapies remain poorly understood. Our analysis revealed significantly diminished microbial richness (Chao and Sobs indices) in refractory cases, despite comparable Shannon diversity across groups (Fig. 6A through C). PCoA analysis revealed substantial structural shifts in the refractory group compared with initial infection cases (Fig. 6D and E). The gut microbial community was dominated by Firmicutes and Bacteroidota, which collectively represented over 50% of the taxonomic composition (Fig. 6F). While commensal bacteria including *Bacteroides*, *Prevotella*, and *Faecalibacterium* were enriched in Group S, some pathogenic taxa including *Streptococcus* and *Enterobacter* were elevated significantly after refractory *H. pylori* infection (Fig. 6G and H).

## DISCUSSION

In this study, we delineated the structure of gastrointestinal microbiome in patients with refractory *H. pylori* infection. Our findings demonstrated that the patients underwent repeated eradication treatments and harbored gastrointestinal microbiota dysbiosis, characterized by reduced microbial diversity. Furthermore, we observed differential microbiota profiles associated with specific antibiotic resistance patterns, with *Burkholderia-Caballeronia-Paraburkholderia, Veillonella*, and *Peptostreptococcus* showing increased relative abundance in refractory cases. The gut microbiota structure was significantly altered following multiple eradication therapies, with a notable proliferation of pathogenic bacterial taxa, particularly *Streptococcus* and *Enterobacter*, observed in refractory individuals.

The gastric microbiota, primarily comprising Proteobacteria, Firmicutes, Bacteroidetes, and Actinobacteria, serves essential functions in gastric homeostasis (24). *H. pylori* infection induces profound microbial restructuring, marked by decreased alpha diversity and expansion of Proteobacteria (the phylum containing *H. pylori*), alongside depletion of Bacteroidetes and Firmicutes (25). This dysbiotic state may facilitate gastric disease pathogenesis. Notably, non-*H. pylori* species, including *Streptococcus anginosus* and *Fusobacterium nucleatum,* have been implicated in gastric carcinogenesis (26, 27). Importantly, *H. pylori* eradication enables partial microbiota recovery, with compositional shifts toward *H. pylori*-negative profiles. This restoration is associated with a reduction in microbial dysbiosis indices. In contrast, patients who fail eradication therapy exhibit persistent microbial imbalances, with reduced diversity and altered bacterial composition (10). Our study further demonstrated that patients who underwent multiple failed eradication rounds had strikingly lower gastric microbiota diversity. Consistent with prior reports (28, 29), refractory *H. pylori* infection was associated with significant microbial shifts, featuring marked expansion of opportunistic pathogens, including *Pseudomonas*, *Acinetobacter,* and concomitant depletion of commensal taxa such as *Bifidobacterium*, *Roseburia*, and *Blautia*. The use of antibiotics that are involved in the eradication regimen has a profound impact on the gastrointestinal microbiota (30). Studies have reported that antibiotic-induced alterations in the gastrointestinal microbiota can persist for weeks to months, with significant reductions in beneficial commensal bacteria, such as *Bacteroides* and *Bifidobacterium* (30). Probiotics *Bifidobacterium* can modulate immune responses and inhibit the colonization of *H. pylori* (31)*. Roseburia* produces butyrate, promoting mucosal integrity and exerting anti-inflammatory effects (32). *Blautia* can enhance microbial diversity and sustain metabolic homeostasis (33). Antibiotic-induced disruptions of beneficial bacteria may weaken their inhibitory effects on pathogenic species, leading to overgrowth of pathogens and contributing to gastric microbiota dysbiosis (25).

Notably, our findings revealed that the relative abundance of pathogenic *Burkholderia-Caballeronia-Paraburkholderia*, *Pseudomonas*, and *Acinetobacter* genera was strikingly higher in refractory *H. pylori*-infected individuals compared with treatment-naïve subjects. Prior reports have demonstrated that antibiotic administration can lead to the elevation of *Burkholderia-Caballeronia-Paraburkholderia*, a bacterial group frequently associated with intrinsic multidrug resistance, consistent with our findings (34, 35). *Burkholderia-Caballeronia-Paraburkholderia*, a Proteobacteria member, shows significant clinical correlation with sepsis-induced cholestasis severity in intra-abdominal infections (36). Experimental studies have revealed that high-dose enrofloxacin treatment induces substantial gastrointestinal microbiota alterations, particularly marked by the proliferation of Proteobacteria, including *Burkholderia*, *Pseudomonas*, and *Acinetobacter*, compared with low-dose or untreated controls (37). These findings align with the work by Diamond et al., who reported a marked expansion in Proteobacteria, particularly *Burkholderiaceae* family members, in amoxicillin-treated mice, with *in vitro* studies confirming *Burkholderia cepacia*’s high resistance to amoxicillin (38). The underlying mechanism may involve *Burkholderia*’s inherent resistance to antimicrobial peptides and Polymyxin B, potentially facilitating the development of resistance in previously susceptible species and creating ecological niches for opportunistic pathogens with high intrinsic resistance (39). Our study also identified a significant enrichment of pathogenic *Pseudomonas* and *Acinetobacter* in refractory cases, which is in accordance with prior research (29). Several species of both *Pseudomonas* and *Acinetobacter* are intrinsically resistant to several classes of antibiotics (40). Additionally, the persistence of *H. pylori* in refractory cases may be attributed to the acquisition of drug resistance through genetic mutations, compounding eradication challenges. Therefore, repeated antibiotic exposure promotes both pathogenic bacterial overgrowth and enhanced antibiotic resistance, further complicating refractory *H. pylori* infection management.

We further compared gastric microbiota composition between antibiotic-resistant and antibiotic-sensitive groups, revealing distinct microbial profiles among patients with different antibiotic resistance patterns. Our analysis demonstrated significantly increased microbial richness in the clarithromycin-resistant group, while no distinct difference in microbial diversity was observed in the levofloxacin-resistant and multidrug-resistant groups relative to the antibiotic-sensitive group. Specifically, we found that *Veillonella* exhibited a significant increase in levofloxacin-resistant patients, while *Micromonospora* was consistently elevated in those with clarithromycin and multidrug resistance. Multiple studies have identified *Veillonella* enrichment within the gastric microbiota of gastric cancer patients, with strong competitive interactions between *Helicobacter* and *Veillonella* observed in advanced gastric lesions (10). *Micromonospora*, known for producing antibiotics, may influence the gastric microenvironment, favoring the persistence of resistant *H. pylori* strains (41, 42). Its presence could either drive antibiotic resistance through selective pressure or thrive in the dysbiotic conditions created by resistant infections. Conversely, its antimicrobial properties might offer therapeutic potential (43), underscoring the need for further exploration of *Micromonospora* as a biomarker or therapeutic target in refractory *H. pylori* infections. Notably, patients with multidrug-resistant infections exhibited significantly elevated relative abundances of *Peptostreptococcus. Peptostreptococcus* has been implicated in gastric carcinogenesis, with *Peptostreptococcus anaerobius* shown to exacerbate colonic epithelial damage, disrupt the intestinal barrier, and induce dysbiosis by reducing beneficial bacteria like *Bifidobacterium* and *Lactobacillus* (44, 45). Furthermore, *Peptostreptococcus stomatis* and *Peptostreptococcus anaerobius* have been associated with colonic tumorigenesis and may influence the efficacy of immune checkpoint blockade therapy (46, 47). Due to varying bacterial susceptibility to antibiotics, patients with different antibiotic resistance profiles exhibit distinct structural characteristics in their gastric microbiota. However, the association between these bacteria and antibiotic resistances requires further validation through *in vitro* experiments.

Previous studies have shown that *H. pylori* eradication can temporarily disrupt the gut microbiota, with gradual recovery over time. However, our study revealed that patients with multiple eradication failures exhibited persistent gut microbiota dysbiosis even 6 months post-treatment, significantly differing from untreated *H. pylori*-infected patients. Fecal microbiome analysis revealed significant alterations in refractory *H. pylori* infection, characterized by decreased *Bacteroides* and *Prevotella* alongside increased *Streptococcus* abundance. *Bacteroides* and *Prevotella,* belonging to Bacteroidetes, are bacteria that produce short-chain fatty acids (SCFAs). As SCFAs modulate inflammatory responses, energy metabolism, and gut homeostasis, their depletion may contribute to disease pathogenesis. Notably, Zhou et al. identified *Streptococcus anginosus* and *S. constellatus* as a sensitive and specific biomarker combination for gastric cancer detection (48). Similarly, *Enterobacter* species, such as *E. cloacae*, are known opportunistic pathogens linked to antibiotic resistance and nosocomial infections (49). These findings align with reports that repeated antibiotic exposure disrupts microbial homeostasis, favoring the proliferation of resistant and pathogenic taxa (50). The persistent enrichment of these genera in refractory *H. pylori* patients underscores the potential for repeated eradication therapies to exacerbate dysbiosis, potentially worsening disease outcomes and complicating future treatment strategies.

Our findings reveal concurrent alterations in gastric and intestinal microbiota among refractory *H. pylori*-infected patients following multiple antibiotic regimens. These perturbations may operate through independent mechanisms rather than representing downstream effects of gastric dysbiosis on the gut ecosystem. First, fundamental anatomic and physiologic barriers exist between gastric and intestinal niches in healthy individuals, characterized by distinct differences in microbial biomass (intestinal >10³-fold higher than gastric), pH gradients, and oxygen availability (51). These compartmentalized environments inherently constrain direct microbial crosstalk. Second, gastric mucosa samples were utilized to analyze colonized bacteria in the stomach, which minimized confounding from transient luminal bacteria. Third, our findings demonstrate site-specific antibiotic susceptibility patterns. The gastric mucosa exhibited enrichment of opportunistic pathogens, such as *Pseudomonas, Burkholderia,* and depletion of commensals, including *Bifidobacterium*, whereas gut microbiota showed an increase of *Streptococcus* and a decrease of *Bacteroides*. While these findings support compartmentalized dysbiosis, systemic effects of antibiotic exposure, such as immune modulation and metabolic shifts, may concurrently influence both sites. Future studies are expected to elucidate whether gastric microbiota-derived signals indirectly modulate distal microbial ecology.

This study has several limitations. First, the baseline data of the samples were not completely matched. Although we employed statistical adjustments to account for these differences, validation of our findings will require larger, well-matched cohorts in future investigations. Second, while this study reveals distinct microbiota profiles between treatment-naïve patients and refractory cases, the retrospective design precludes definitive causal inference regarding whether the microbial dysbiosis precedes or results from antibiotic resistance. Future prospective studies tracking microbiota dynamics before, during, and after antibiotic therapy are warranted to clarify whether gut dysbiosis is a cause or consequence of resistance development. This would also help identify predictive biomarkers for treatment failure. Third, the utilization of 16S rRNA amplicon sequencing, while offering cost-effectiveness and analytical simplicity, is limited to taxonomic profiling and cannot provide functional or genomic information. Additionally, potential PCR amplification biases may affect sequence representation. Although we previously attempted metagenomic sequencing for gastric mucosa samples, the results were substantially confounded by host DNA contamination. Future studies should incorporate sample pretreatment protocols prior to metagenomic sequencing to enhance bacterial identification accuracy and explore microbial functional profiles. Moreover, although the collection of fecal samples is non-invasive and convenient, it includes both resident and transient bacteria, which may not fully represent mucosal-colonizing microbiota. Subsequent studies could incorporate intestinal mucosal biopsies for more precise analysis. Finally, the differential bacteria identified through sequencing were not validated using qPCR, which should be addressed in future research.

### Conclusion

Due to repeated eradication medications, refractory *H. pylori*-infected patients experienced persistent gastrointestinal microbiota dysbiosis, characterized by reduced diversity, depletion of beneficial bacteria (e.g., *Bifidobacterium*, *Roseburia*), and overgrowth of pathogens (e.g., *Burkholderia*, *Streptococcus*). Notably, antibiotic resistance patterns, such as levofloxacin or clarithromycin resistance, differently shape gastric microbiota composition. Therefore, improving first-line eradication success is critical to avoid repeated treatments, antibiotic resistance, and gastrointestinal dysbiosis. Given the observed microbiota imbalances in refractory cases, adjunctive probiotic supplementation may be considered to enhance eradication efficacy in subsequent therapies.

## Data Availability

The raw Illumina sequencing data have been deposited in the NCBI Sequence Read Archive (SRA, http://www.ncbi.nlm.nih.gov/sra) under accession number PRJNA801428 for gastric mucosa samples and PRJNA801350 for fecal samples.

## SUPPLEMENTAL MATERIAL

Table S1 msystems.01090-25-s0001.docx Clinical baseline data between treatment-naïve patients with *H. pylori* infection and patients with refractory *H. pylori* infection.

Table S2 msystems.01090-25-s0002.docx Clinical baseline data between clarithromycin-susceptible and clarithromycin-resistant patients.

Table S3 msystems.01090-25-s0003.docx Clinical baseline data between levofloxacin-susceptible and levofloxacin-resistant patients.

Table S4 msystems.01090-25-s0004.docx Clinical baseline data between single drug and multidrug-resistant patients.

Table S5 msystems.01090-25-s0005.docx Specific explanation of functional prediction pathway.

ASM does not own the copyrights to Supplemental Material that may be linked to, or accessed through, an article. The authors have granted ASM a non-exclusive, world-wide license to publish the Supplemental Material files. Please contact the corresponding author directly for reuse.
