# Supplementary material for: Comprehensive characterization of gastrointestinal microbiota dysbiosis in patients with refractory Helicobacter pylori infection
Source: mSystems. 2025 Sep 30;10(10):e01090-25. doi: 10.1128/msystems.01090-25 (PMC12542668; doi:10.1128/msystems.01090-25)
Supplement: Table S1 — Clinical baseline data between treatment-naïve patients with H. pylori infection and patients with refractory H. pylori infection. [file msystems.01090-25-s0001.docx]

Table S1. Clinical baseline data between treatment-naïve patients with *H. pylori* infection and patients with refractory *H. pylori* infection. S, treatment-naïve patients with *H. pylori* infection; F, patients with refractory *H. pylori* infection; BMI, body mass index.

|  | | S | F | *P* value |
| --- | --- | --- | --- | --- |
| Number | | 32 | 84 | / |
| Age (year) | | 40.41 ± 10.89 | 46.63 ± 11.27 | 0.009 |
| Sex (female, %) | | 22 (68.75%) | 39 (46.43%) | 0.068 |
| BMI (Kg/m^2^) | | 21.28 ± 2.40 | 22.75 ± 2.92 | 0.050 |
| Antimicrobial Susceptibility | Metronidazole | / | 56 (96.55%) | / |
|  | Clarithromycin | / | 45 (77.59%) | / |
|  | Levofloxacin | / | 33 (56.90%) | / |
|  | Rifampicin | / | 3 (5.17%) | / |
|  | Tetracycline | / | 1 (1.72%) | / |
|  | Amoxicillin | / | 0 (0) | / |
|  | Furazolidone | / | 0 (0) | / |
| Dual drug resistance | | / | 16 (27.59%) | / |
| Multidrug resistance | | / | 32 (55.17%) | / |
