# Supplementary material for: Comprehensive characterization of gastrointestinal microbiota dysbiosis in patients with refractory Helicobacter pylori infection
Source: mSystems. 2025 Sep 30;10(10):e01090-25. doi: 10.1128/msystems.01090-25 (PMC12542668; doi:10.1128/msystems.01090-25)
Supplement: Table S2 — Clinical baseline data between clarithromycin-susceptible and clarithromycin-resistant patients. [file msystems.01090-25-s0002.docx]

Table S2. Clinical baseline data between clarithromycin susceptible and resistant patients. Cla_S, patients with clarithromycin susceptibility; Cla_R, patients with clarithromycin resistance.

|  | Cla_S | Cla_R | *P* value |
| --- | --- | --- | --- |
| Number | 13 | 45 | / |
| Age (year) | 43.62 ± 15.20 | 46.89 ± 10.37 | 0.477 |
| Sex (female, %) | 6 (46.15%) | 26 (57.78%) | 0.467 |
