# Supplementary material for: Comprehensive characterization of gastrointestinal microbiota dysbiosis in patients with refractory Helicobacter pylori infection
Source: mSystems. 2025 Sep 30;10(10):e01090-25. doi: 10.1128/msystems.01090-25 (PMC12542668; doi:10.1128/msystems.01090-25)
Supplement: Table S3 — Clinical baseline data between levofloxacin-susceptible and levofloxacin-resistant patients. [file msystems.01090-25-s0003.docx]

Table S3. Clinical baseline data between levofloxacin susceptible and resistant patients. Lev_S, patients with levofloxacin susceptibility; Lev_R, patients with levofloxacin resistance.

|  | Lev_S | Lev_R | *P* value |
| --- | --- | --- | --- |
| Number | 25 | 33 | / |
| Age (year) | 43.04 ± 13.09 | 48.52 ± 9.80 | 0.087 |
| Sex (female, %) | 15 (60.00%) | 17 (51.52%) | 0.528 |
