# Supplementary material for: Comprehensive characterization of gastrointestinal microbiota dysbiosis in patients with refractory Helicobacter pylori infection
Source: mSystems. 2025 Sep 30;10(10):e01090-25. doi: 10.1128/msystems.01090-25 (PMC12542668; doi:10.1128/msystems.01090-25)
Supplement: Table S4 — Clinical baseline data between single drug and multidrug-resistant patients. [file msystems.01090-25-s0004.docx]

Table S4. Clinical baseline data between single drug and multidrug resistant patients. Multi_R, patients with multidrug resistance; Sig_R, patients with single drug resistance.

|  | Multi_R | Sig_R | *P* value |
| --- | --- | --- | --- |
| Number | 29 | 10 | / |
| Age (year) | 48.90 ± 8.99 | 41.80 ± 15.04 | 0.186 |
| Sex (female, %) | 15 (51.72%) | 4 (40.00%) | 0.543 |
