# Supplementary material for: Comprehensive characterization of gastrointestinal microbiota dysbiosis in patients with refractory Helicobacter pylori infection
Source: mSystems. 2025 Sep 30;10(10):e01090-25. doi: 10.1128/msystems.01090-25 (PMC12542668; doi:10.1128/msystems.01090-25)
Supplement: Table S5 — Specific explanation of functional prediction pathway. [file msystems.01090-25-s0005.docx]

Table S5. Specific explanation of functional prediction pathway.

| KO ID | Explanation |
| --- | --- |
| ko00052 | Metabolism; Carbohydrate metabolism; Galactose metabolism |
| ko00121 | Metabolism; Lipid metabolism; Secondary bile acid biosynthesis |
| ko00330 | Metabolism; Amino acid metabolism; Arginine and proline metabolism |
| ko00510 | Metabolism; Glycan biosynthesis and metabolism; N-Glycan biosynthesis |
| ko00511 | Metabolism; Glycan biosynthesis and metabolism; Other glycan degradation |
| ko00520 | Metabolism; Carbohydrate metabolism; Amino sugar and nucleotide sugar metabolism |
| ko00550 | Metabolism; Glycan biosynthesis and metabolism; Peptidoglycan biosynthesis |
| ko00600 | Metabolism; Lipid metabolism; Sphingolipid metabolism |
| ko00680 | Metabolism; Energy metabolism; Methane metabolism |
| ko00710 | Metabolism; Energy metabolism; Carbon fixation in photosynthetic organisms |
| ko00900 | Metabolism; Metabolism of terpenoids and polyketides; Terpenoid backbone biosynthesis |
| ko00943 | Metabolism; Biosynthesis of other secondary metabolites; Isoflavonoid biosynthesis |
| ko00980 | Metabolism; Xenobiotics biodegradation and metabolism; Metabolism of xenobiotics by cytochrome P450 |
| ko00983 | Metabolism; Xenobiotics biodegradation and metabolism; Drug metabolism - other enzymes |
| ko01051 | Metabolism; Metabolism of terpenoids and polyketides; Biosynthesis of ansamycins |
| ko04113 | Cellular Processes; Cell growth and death; Meiosis - yeast |
| ko04141 | Genetic Information Processing; Folding, sorting and degradation; Protein processing in endoplasmic reticulum |
| ko04974 | Organismal Systems; Digestive system; Protein digestion and absorption |
| ko00312 | Human Diseases; Drug resistance: antimicrobial;beta-Lactam resistance |
| ko00120 | Metabolism; Lipid metabolism; Primary bile acid biosynthesis |
